# Uplifting Behavior of Shallow Buried Pipe in Liquefiable Soil by Dynamic Centrifuge Test

**DOI:** 10.1155/2014/838546

**Published:** 2014-07-10

**Authors:** Bo Huang, Jingwen Liu, Peng Lin, Daosheng Ling

**Affiliations:** ^1^MOE Key Laboratory of Soft Soils and Geoenvironmental Engineering, Department of Civil Engineering, Zhejiang University, Hangzhou, 310058, China; ^2^State Key Laboratory of Hydroscience and Engineering, Tsinghua University, Beijing 100084, China

## Abstract

Underground pipelines are widely applied in the so-called lifeline engineerings. It shows according to seismic surveys that the damage from soil liquefaction to underground pipelines was the most serious, whose failures were mainly in the form of pipeline uplifting. In the present study, dynamic centrifuge model tests were conducted to study the uplifting behaviors of shallow-buried pipeline subjected to seismic vibration in liquefied sites. The uplifting mechanism was discussed through the responses of the pore water pressure and earth pressure around the pipeline. Additionally, the analysis of force, which the pipeline was subjected to before and during vibration, was introduced and proved to be reasonable by the comparison of the measured and the calculated results. The uplifting behavior of pipe is the combination effects of multiple forces, and is highly dependent on the excess pore pressure.

## 1. Introduction

Pipelines are the artery of modern industries and urban life, widely used in lifeline engineerings such as water supply, electricity supply, natural gas transportation line, and communication cables. According to a large number of seismic disaster surveys [1, 2], the damage probability of the underground pipelines in liquefied soil is far greater than that in the nonliquefied soil. Shallow buried pipelines in liquefied sands might float upward displaying deflection deformation, and sometimes they even go above the ground, which often aggravates the damage degree. In the 1964's Niigata earthquake, among a total pipeline length of 470 kilometers, 68% of pipelines were destroyed. The pipelines located under water were damaged particularly seriously, whose failures were mainly due to uplifting deformation. Hereafter, “uplifting” phenomena of underground pipelines and other underground structures were more and more frequently observed in seismic incidents, such as the Loma Prieta earthquake and the Nansei-Oki earthquake [3–6].

There have been quite a lot of studies concentrating on the “uplifting” phenomenon of pipelines during soil liquefaction processes. The factors that affect the stress and deformation of pipelines during floating; for example, the buried depth, diameter, thickness, stiffness of the pipelines, the type, liquefied area, stiffness, and strength of soil, have been studied [7, 8]. It is generally considered that the diameter of pipeline, liquefied depth, stiffness of soil are critical parameters which have great effects on the structure deformation of buried pipeline; the largest floating displacement occurs in the central of the pipe. Some experts made a number of numerical and experimental researches on the response differences between the free field and field with pipelines during seismic vibration. Kitaura et al. [9] developed a hybrid procedure to study the pipeline response in the liquefied field. The numerical model shows that the pipeline response to seismic vibration is more significant when the excess pore pressure is low, and the buoyancy force increases with increasing excess pore pressure. Ling et al. [10] conducted centrifuge tests to investigate the seismic response differences of free field and field with pipelines with respect to the acceleration, excess pore pressure and settlement of ground surface, and so forth. Other experts [11, 12] investigated the effects of dilatancy angle and relative density of soil, diameter and buried depth of pipeline, underground water table level and thickness of the saturated soil layer, and so forth, on the uplifting behavior of pipeline, which indicated that the buried depth of pipe had the most significant impact.

Despite that the above mentioned studies have investigated the pipeline uplifting behaviors in liquefied fields with respect to influencing factors involving the pipe itself and the soil properties, through a comprehensive means of field investigations, numerical simulations, and model tests, there still exists disagreement on the understanding of the mechanism of pipeline uplifting. Some [13, 14] believe that the uplifting of pipelines is associated with the loss of soil shear strength due to soil liquefaction. Others [15] come to a conclusion that uplifting is simply related to the vibration rather than soil liquefaction, based on the observed phenomenon that uplifting starts when the soil is not fully liquefied, and ceases when the shaking is finished even when the excess pore pressure is still very high. Due to lack of understanding of the pipeline uplifting mechanism, different approaches are adopted to calculate the buoyancy force that pipelines are subjected to during soil liquefaction. In general, it is calculated using the formula *F*_Buoyancy_ = *ρ*_sat⁡_ *gV*, where the saturated soil is considered as fluidized material having a unit weight equivalent to its saturated unit weight [16–18]. It is worth mentioning that the buoyancy force is estimated in terms of excess pore pressure as well [19].

It is significantly favorable that researches are made on the stress conditions of the pipelines during uplifting for an in-depth understanding of the mechanism of pipe uplifting behaviors. In the present study, dynamic centrifuge model tests were conducted to investigate the mechanism of pipeline uplifting phenomenon during soil liquefaction. Based on the measurements of acceleration, excess pore pressure and earth pressure around the pipelines, the forces on the pipelines before and during soil liquefaction were estimated, and the mechanism and the main influencing factors of pipeline uplifting were analyzed.

## 2. Test Equipment and Programs

### 2.1. Centrifuge, Shaking Table, and Rigid Container

**Figure 1:**
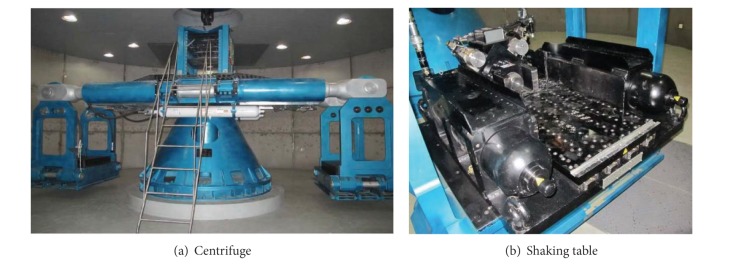
Centrifuge and shaking table.

The tests are conducted on the ZJU400 centrifuge with a shaking table, shown in Figure 1. The beam type centrifuge, with a payload capacity of 400 gt, has double platforms and an effective arm radius of 4.5 m. The maximum centrifugal acceleration is 100 g for dynamic tests. The centrifuge platforms have an overall dimension of 1.5 m × 1.2 m × 1.5 m. Meanwhile, an in-flight uniaxial electrohydraulic shaking table has been made to simulate seismic excitation. The shaking table has vibration frequencies ranging from 10 Hz~200 Hz. Its payload capacity is 500 kg, and its maximum lateral displacement and acceleration are 0.6 cm and 40 g, respectively. More details about the device can be found in [20].

A Rigid container was used to prepare the model, whose inner dimension is 0.6 m (length) × 0.4 m (width) × 0.5 m (height), and its front perspex made window is convenient for direct observation of the experimental phenomena. A 25 mm thick piece of mouldable Duxseal was placed on each side of the container to reduce reflecting incident stress waves by at least 65% [21].

### 2.2. Model Pipe

The model pipes are made of aluminum tube, with a density of 2.7 g/cm^3^, a length of 390 mm, and an inner and outer diameter of 36 mm and 40 mm, respectively. And they are used to simulate large diameter pipes like oil or gas pipelines. Each end of the pipes was sealed by a perspex disc with PTFE, and petroleum jelly was also used to reduce end friction. Microearth pressure transducers were installed on the bottom, side and crown of pipes to measure earth pressures (the normal stress).

Two model pipes were buried in the ground. One was used for measuring the uplift displacement, named pipe 1#; the other was used for measuring the stabilizing force during soil liquefaction, named pipe 2#. Pipe 1# could move freely during tests. Pipe 2# was installed to the rigid container through a connecting rod. The force, provided by the connecting rod to keep pipe 2# stable in the vertical direction, was defined as stabilizing force. In order to acquire the stabilizing force of pipe in the centrifuge, a load cell was installed on the connecting rod.

**Figure 2:**
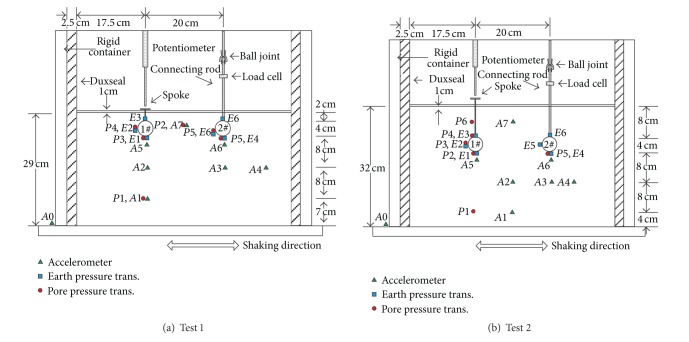
The layout of pipes and sensors.

In order to measure vertical displacements of underground structures in the centrifuge, draw-wire displacement potentiometers are commonly used [22]. However, the potentiometer cable has tension force which will reduce the structure's self-weight to some extent. Moreover, the tension force varies with the centrifugal acceleration, which is hard to be calibrated. Therefore, two aluminum alloy spokes with discs on the end were installed on pipe 1#, as shown in Figure 2. The vertical displacements of the discs could be measured by potentiometers, which guaranteed a more precise and reliable measurement while pipe 1# moved freely.

A simple device of a connecting rod with ball joint was developed, as shown in Figure 2. This device kept pipe 2# stable in the vertical direction, and meanwhile moved freely in the horizontal direction. As pipe 2# was stable in the vertical direction, the shear strength of the overlaying soil would not take a part in the measured data of the force that pipe 2# is subjected to during vibration.

### 2.3. Sand and Viscous Fluid

Fujian standard sand, which is widely used in China for geotechnical physical modeling tests [23, 24], was adopted in the present study. It has a mean diameter (*D*_50_) 0.16 mm; the uneven coefficient (*C*_*u*_) and the curvature coefficient (*C*_*c*_) are 1.6 and 0.95, respectively. The maximum and the minimum void ratios are 0.96 and 0.61, respectively. The model foundation was prepared by pluviation method. The sand was rained from a sieve in a hopper into the container, where the falling height of the hopper and the shape of the sieve were kept unchanged based on the precalibrated results to obtain a constant relative density. The designed relative density of the two tests was 60%. The heights of the model foundation were 29 cm and 32 cm in test 1 and test 2, respectively.

There is a conflict between dynamic and permeability time scale, for the former is 1/*n*, and the latter is 1/*n* ^2^. To solve the problem, viscous fluid was introduced to reduce the permeability of soil. Methyl cellulose fluid, which is commonly used in geotechnical centrifuge modeling tests, has similar compressibility and density to water [25]. Furthermore, it is also capable of sustaining high pore pressure for liquefaction studies and can have any viscosity by changing the mixture ratio of hydroxypropyl methylcellulose (HPMC) powder to water. Based on the precalibrated relationship of mixture ratio, permeability, and temperature, the mixture ratio was determined to be 3.0% at a centrifugal acceleration 30 g. Methyl cellulose fluid was prepared in water with a temperature of 70°C and introduced at a rate of 0.1 L/h into the model foundation when cooling down, which was slow enough to avoid sand boil phenomenon. The vacuum method is chosen to saturate the soil, the whole process of saturation took over 200 h and the water level was kept 1 cm above the ground when the saturation is completed.

### 2.4. Seismic Excitations

Three types of excitation waves were adopted, that is, EL-Centro wave, Taft wave, and Zhejiang seism wave. El-Centro wave was recorded in the Imperial Valley earthquake of California in 1940, with a primary period of 0.5 s, belonging to near earthquake. Taft wave was recorded in the earthquake happened in Kern of California in 1952, with a primary period of 0.5 s, belonging to distant earthquake. These two waves are commonly used. Zhejiang seism wave is an artificial seismic wave suited the seismic zoning type of Zhejiang Province in China, with a 10 s duration. Assuming the exceeding probability of Zhejiang seism wave to be 10% and 2%, the maximum acceleration is 63.1 cm/s^2^ and 153 cm/s^2^, respectively.

### 2.5. Testing Procedures

In the present study, two centrifuge tests were conducted on pipes of different buried depths under the same ground conditions. The centrifuge accelerations were both 30 g. The buried depths, measured from the top of pipes to the ground surface, were 20 mm (equal to 0.5*D*) for test 1 and 80 mm (equal to 2*D*) for test 2. Accelerometers, pore pressure transducers, earth pressure transducers, potentiometers, and load cell were used in the tests [26]. The layout of the sensors and pipes for each test is shown in Figure 2.

When started, the centrifuge was accelerated to 30 g gradually. The relative densities of the ground before shaking were 65.2% for test 1 and 61.9% for test 2. The excitation progress was divided into 3 stages based on the acceleration amplitudes from weak to strong. White noise excitations were applied before and after each stage to test the dynamic characteristics of the model. The schedule of excitations as well as the uplifting status of the pipe 1# at each shaking stage is shown in Table 1. There was at least a 30 min interval between two shaking stages, so that the excess pore pressure can dissipate entirely. After all the excitations were applied, the test data as will be mentioned in the following sections are converted to the prototype scale.

## 3. Results of Tests

### 3.1. The Degrees of Soil Liquefaction and the Pore Pressure Response around the Pipe

#### 3.1.1. The Degrees of Soil Liquefaction

**Figure 3:**
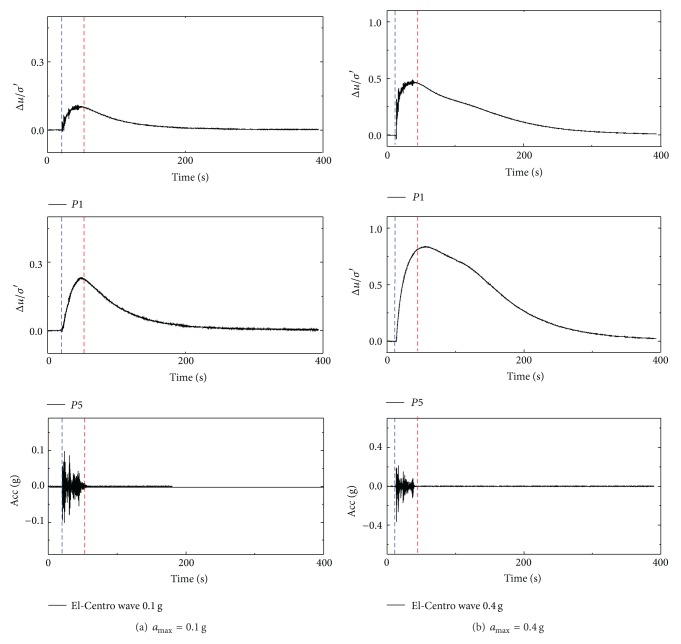
Ground response of Δ*u*/*σ*′ in test 2 under El-Centro wave.

The excess pore pressure ratio Δ*u*/*σ*′ defined as the value of the excess pore pressure normalized by the initial vertical effective stress represents the degree of soil liquefaction. If the value of Δ*u*/*σ*′ reaches one, it means the soil is fully liquefied. As the build-up of excess pore pressure in the two tests was similar, only part of the results is given, that is, the variations of Δ*u*/*σ*′ in test 2 under 0.1 g and 0.4 g excited by El-Centro wave, as shown in Figure 3. The buried depths of P1 and P5 were 8.4 m and 3.6 m, respectively. It shows that the excess pore pressure generated from the start of the earthquake vibration. As soon as the vibration stopped, the excess pore pressure ratio began to dissipate. In the two tests, although no excitations led the soil to fully liquefied state, the “uplifting” phenomenon still existed, which suggests that there is a high potential for the occurrence of pipe uplifting in incompletely liquefied soil.

#### 3.1.2. The Pore Pressure Response around the Pipe

**Figure 4:**
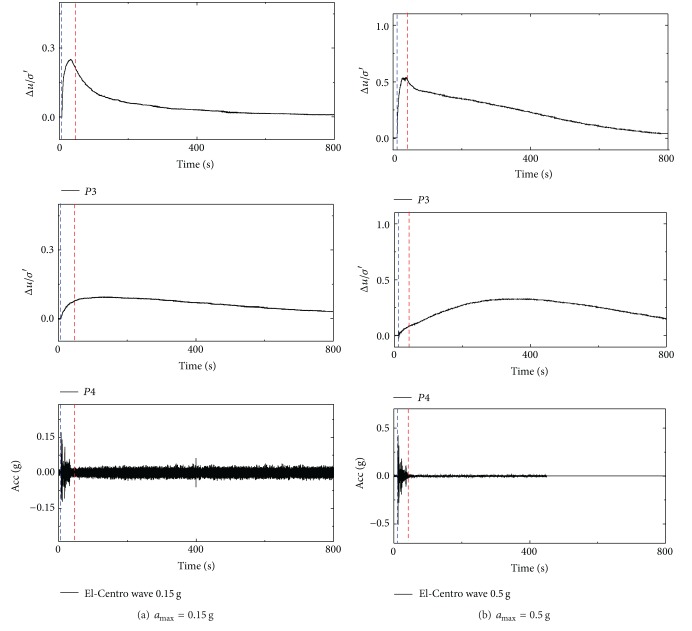
Pipe response of Δ*u*/*σ*′ in test 1 under El-Centro wave.

**Figure 5:**
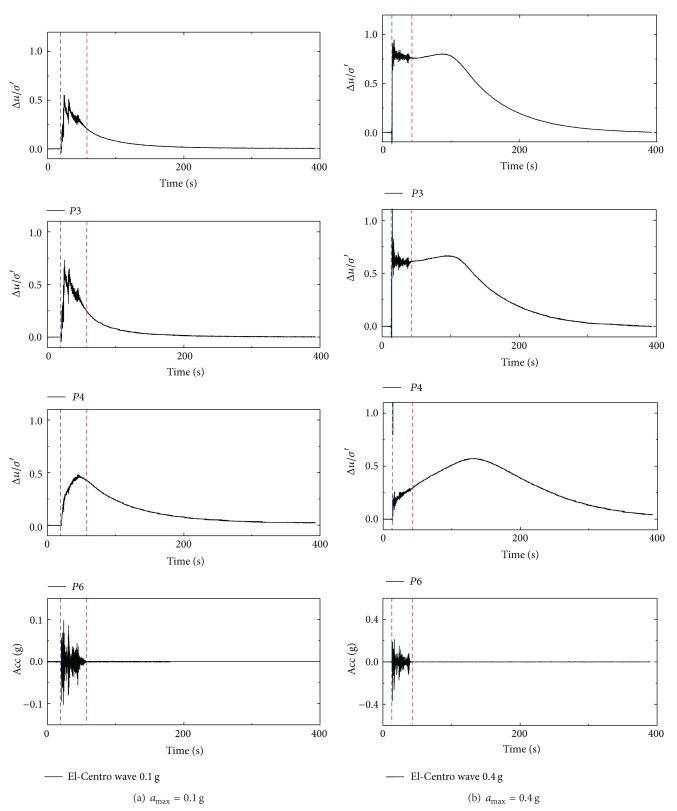
Pipe response of Δ*u*/*σ*′ in test 2 under El-Centro wave.

The variations of Δ*u*/*σ*′ around the pipe in test 1 under 0.15 g and 0.5 g excited by El-Centro wave are shown in Figure 4. P3 and P4 were fixed at the bottom and side of pipe 1#, 1.8 m and 1.2 m below the surface, respectively. As the buried depth of pipes in test 1 was so shallow that the pore pressure could not be measured well at the crown of pipe; therefore, no transducer was installed there. In test 2, transducers P2, P3, and P4 were installed at the bottom, side, and crown of pipe 1#, respectively. P6 was embedded in the soil layer overlying pipe 1#. The variations of Δ*u*/*σ*′ for P3, P4, and P6 under 0.1 g and 0.4 g excited by El-Centro wave are given in Figure 5. Due to the damage of P2, there is no measured data from P2.

It can be figured out from Figures 4 and 5 that excess pore pressures around the pipes respond rapidly once the earthquake load is applied. For a small amplitude excitation, the excess pore pressure dissipates gradually when the excitation ends. Considering that the amplitude of real earthquake wave decreases obviously at the late stages, the excess pore pressures might even dissipate before the end of vibration, as is shown in Figure 5(a). For stronger seismic excitations, the excess pore pressures of soil below the pipe dissipate when the vibration is ceased, but the dissipation rate slows down obviously, as shown in Figure 4(b). Meanwhile, at the side and crown of pipe, the excess pore pressures retains for a while after the vibration is ceased. This phenomenon is attributable to the supply of the pore fluid draining from the base of the rigid container, which was more sufficient than the dissipation of the excess pore pressures at shallow location, so that the excess pore pressures at shallow places keep generating, as shown in Figure 4(b) (location P4) and Figure 5(b) (location P6).

It can be seen that the stronger the excitation is, the larger the excess pore pressures ratio will be. The dissipation rate of the excess pore pressure decreases with the decreasing buried depth of the pipe. And in some cases, the excess pore pressures even keep generating. The frictional resistance between soil grains was largely reduced by the increase in pore pressure. Therefore, pipe floats upward more easily through the soil on the condition of stronger excitations or lower buried depths of pipe.

### 3.2. Earth Pressure Response

**Figure 6:**
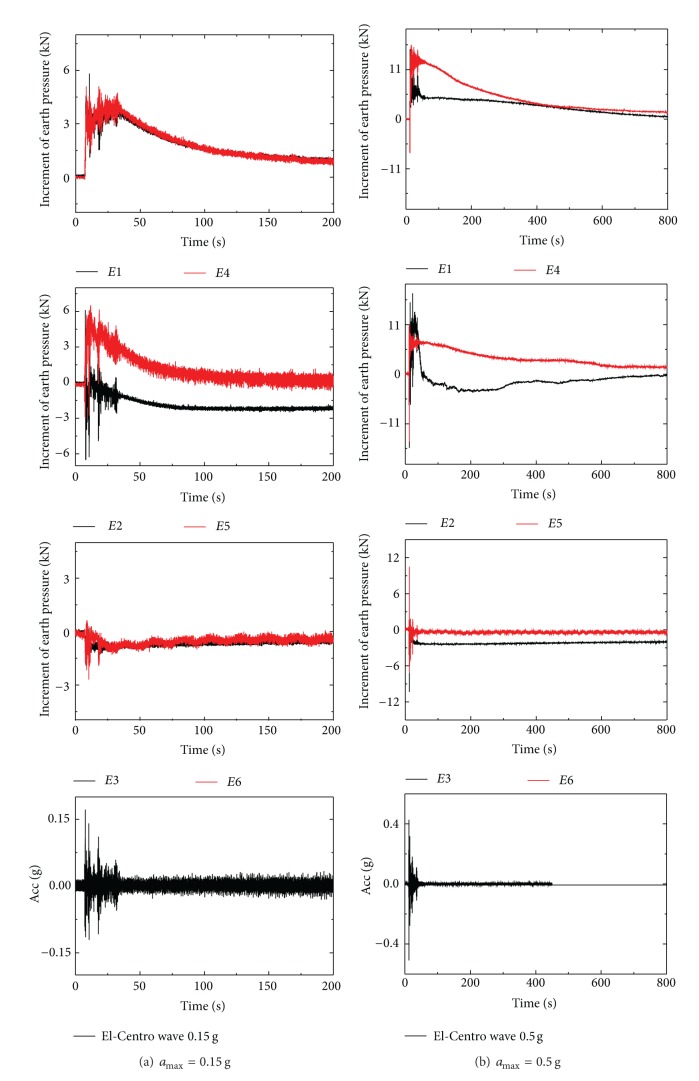
Increment of earth pressure in test 1 under El-Centro wave.

The layout of the earth pressure transducers is shown in Figure 2. And the earth pressure (the total stress, which contains both effective stress and pore pressure) around the circumference of pipe under 0.15 g and 0.5 g excited by EI-Centro wave in test1 is shown in Figure 6. The value of earth pressure changed while the vibration was activated and recovered gradually to 0 after the vibration ended. The responses of the earth pressure were different between pipe 1# and pipe 2# due to the different constraint conditions. Responses of pipe 1# which could move freely were larger than that of pipe 2# which was fixed in the vertical direction. It can be seen in Figure 6 that the increments of the earth pressure at the bottom and side of pipe are proportional to the amplitude of the excitations. However, response of the earth pressure at the crown was weak or even showed a slight decrease, which might indicate the weight reduction of the overlay soil.

### 3.3. Pipe Uplifting

**Figure 7:**
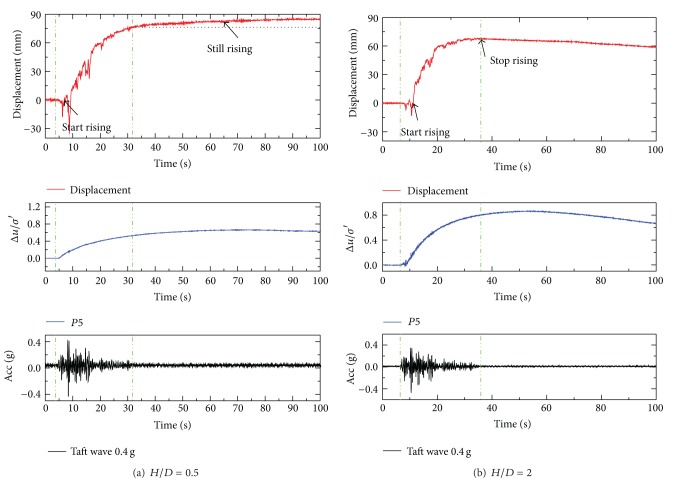
Uplift displacement of pipe 1# at different depth under Taft wave.

**Figure 8:**
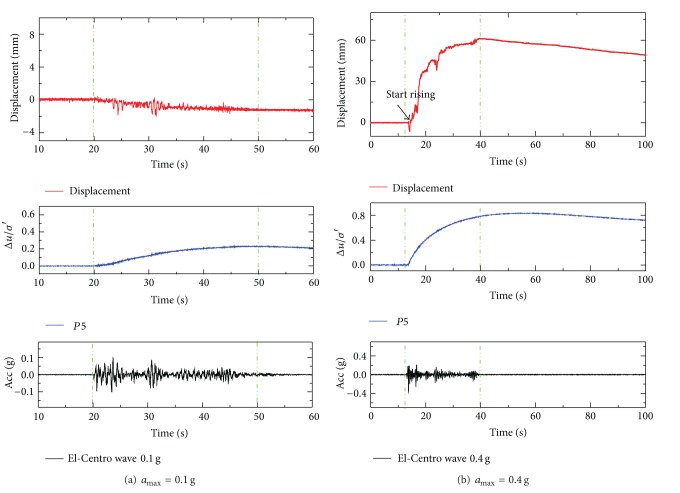
Uplift displacement of pipe 1# in test 2 under El-Centro wave.

It is found that pipeline uplifting takes place once the vibration starts and ceases when the vibration stops despite the presence of high excess pore pressures. Some researchers hold the view that the uplifting of the pipe is highly dependent on the input earthquake motion and weakly related to the increase of excess pore water pressure [15, 19].  Figure 7 shows uplifting responses of pipe 1# under Taft wave in test 1 and test 2. And uplifting responses of pipe 1# under different amplitudes of EL-Centro wave are shown in Figure 8.

**Figure 9:**
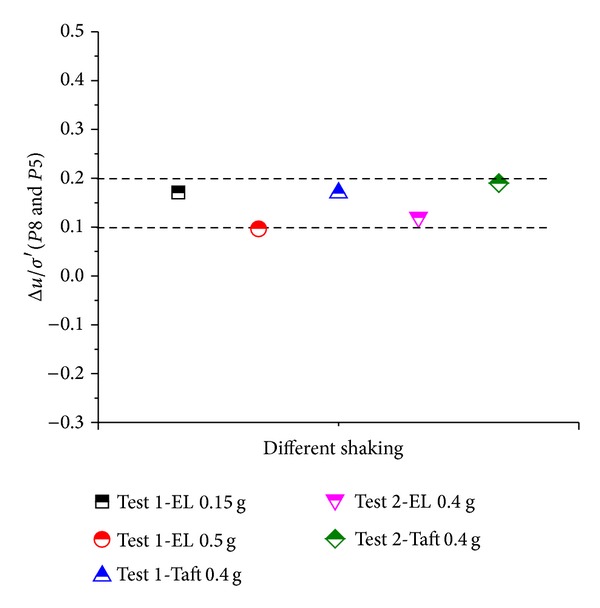
Excess pore pressure of P5 when pipe 1# starts to float.

It is seemingly that the uplifting phenomenon of pipe occurred after shaking and ceased when the shaking ceases. Nevertheless, the uplifting movement is not directly determined by the shaking itself but the response of the pore pressure and the soil pressure. It can be seen that the uplifting of the pipe takes place only after considerable excess pore pressure is generated, rather than immediately after the vibration started. The excess pore pressure ratios of P5 (which were at the same depth of the bottom of pipe 1#) when pipe 1# began to move up were shown in Figure 9. The excess pore pressure ratios distributed between 0.1 and 0.2. However, it does not mean that the pipe will float upward once the excess pore pressure ratio has reached 0.2. As can be seen in Figure 8(a), the pipe settled along with the soil particles under 0.1 g excited by EL-Centro wave, although the maximum excess pore pressure ratio was above 0.2.

Actually, the maximum uplift displacement was not present right after the vibration stopped each time. It can be seen in Figure 7(a), the tendency of uplifting still existed when the earthquake ceased. The uplifting behavior of pipe in liquefied soil is a multiforce coupled behavior, which is not only dependent on the build-up of excess pore pressure but also determined by the shear strength of the soil, relative displacement of pipe and soil, the amplitude of the input seismic wave, and so forth.

### 3.4. The Response of Stabilizing Force

**Figure 10:**
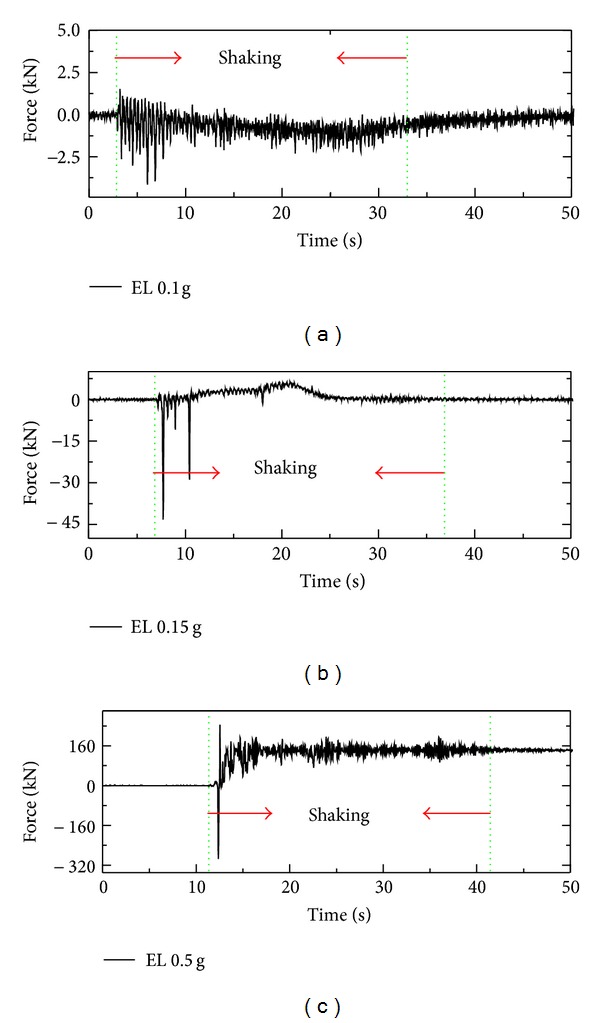
Increment of stabilizing force of pipe 2# under El-Centro wave in test 1.

The stabilizing force, which kept the pipe 2# stable in the vertical direction, was measured by a load cell fixed on the device. The variation of stabilizing force in test 1 under El-Centro wave with different amplitudes is shown in Figure 10. Herein, negative values of the stabilizing force mean that the pipe is prone to settle down, as seen in Figure 10(a), and positive values represents that the pipe has the tendency to uplift, as shown in Figures 10(b) and 10(c).

**Figure 11:**
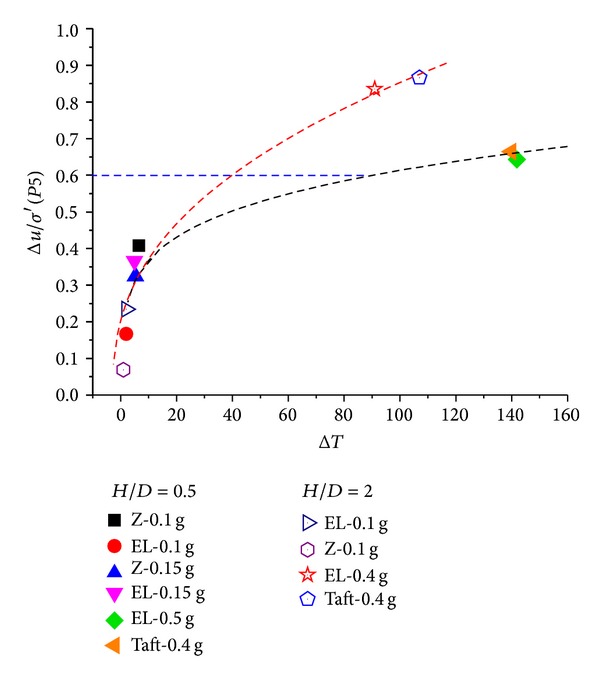
Relationship among increment of stabilizing force, Δ*u*/*σ*′ and excitation.

Figure 11 gives the relationship between the stabilizing force and the excess pore pressure. The abscissa is the stabilizing force at the end of shaking for all tests, and the ordinate is the maximum value of P5. The stabilizing force shows a power function relationship with excess pore pressure. It can be seen that the stabilizing force is larger while the buried depth of the pipe is shallower at the same excess pore pressure ratio. It is probably because that the lateral constraint pressure of soil at shallow depth is smaller than that in deep place, so that the deflection deformation of shallower soil layer can be more intense which makes the pipe uplift more easily.

## 4. Analysis of the Force of Pipe

The force components acting on pipes were investigated with the results obtained from centrifuge tests in this section. These components were adapted from static analysis to a dynamic condition where soil liquefaction occurs. Furthermore, the force analysis was validated by the comparison between the measured and calculated data.

### 4.1. Force Analysis before Shaking

**Figure 12:**
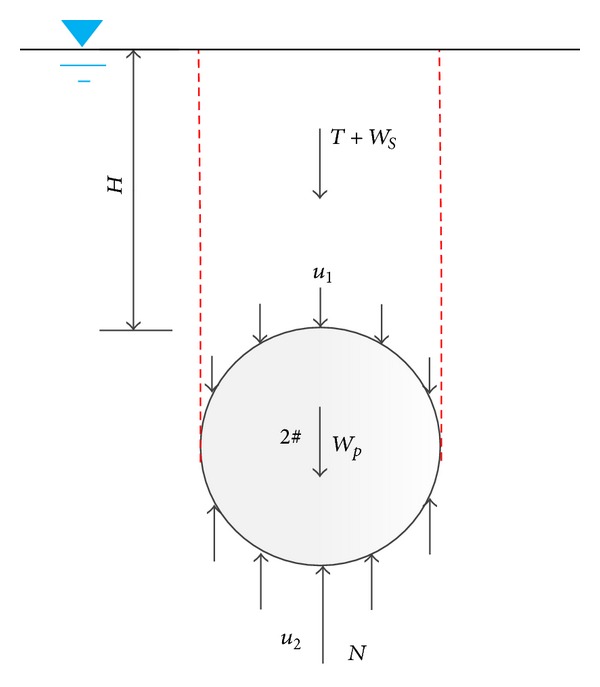
Schematic diagram of force of pipe 2# before shaking.

The force state before shaking is shown in Figure 12. And force equilibrium equation is expressed as follows:

(1) $$\begin{array}{c} T+W_{s}+\overset{D/2}{\underset{-D/2}{\int }}\left(u_{1}\right)dD\cdot L+W_{p}=\overset{D/2}{\underset{-D/2}{\int }}\left(u_{2}\right)dD\cdot L+N, \end{array}$$

where *L* and *W*_*p*_ are the length and weight of the pipe, respectively, which can be obtained according to scaling principle from the length and density of pipe in 1 g condition.* T *is the stabilizing force of pipe 2# which can be measured by load cells (*T* of pipe 1# is 0). *W*_*s*_ is the effective weight of overlying soil;* N *is the support force from the soil underlying the pipe; *u*_1_ and *u*_2_ are pore pressures around the pipe. The total force of these three parts can be calculated by the integral of the earth pressure difference between the upper and the bottom of pipe. It can be written as

(2) $$\begin{array}{c} P=\overset{D/2}{\underset{-D/2}{\int }}\left(u_{2}-u_{1}\right)dD\cdot L+N-W_{s}, \end{array}$$

where *P* denoted the integral of earth pressure difference, which can be obtained by the interpolation according to the earth pressure transducers distributed on the pipes, defined as (3). Consider

(3) $$\begin{array}{c} P=\overset{D/2}{\underset{-D/2}{\int }}\left(e_{2}-e_{1}\right)dD\cdot L, \end{array}$$

where *e*_1_ and *e*_2_ denote the vertical component of linearized earth pressure distributed on the upper half and on the invert half of the pipe, respectively. The earth pressure is the total stress, which contains both effective stress and pore water pressure. The earth pressure mentioned below has the same meaning.

Equation (2) is checked based on the data measured before shaking, which proved to be reasonable with only a little bit difference as stresses around the pipe are estimated based on the interpolating method.

It should be noted that the integral of the pore pressure around the pipe under static state is buoyancy force, which is the static buoyancy force in the present study and can be calculated based on Archimedes principle as follows:

(4) $$\begin{array}{c} \overset{D/2}{\underset{-D/2}{\int }}\left(u_{2}-u_{1}\right)dD\cdot L=\rho_{w}gV_{\text{pipe}}. \end{array}$$

### 4.2. Force Analysis during Shaking for Pipe 2#

**Figure 13:**
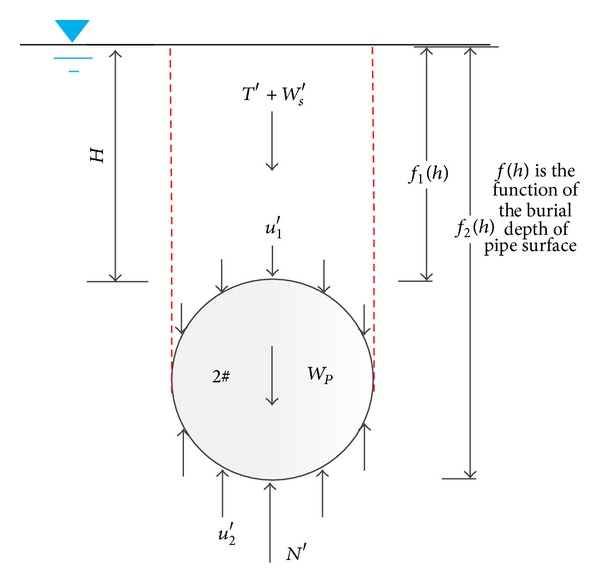
Schematic diagram of force of pipe 2# during shaking.

As pipe 2# was fixed to the rigid container, no displacement in the vertical direction occurred during vibration. And consequently the shear strength of soil could not excite. The force state of pipe 2# during vibration is shown in Figure 13. And force equilibrium equation is expressed in as follows:

(5) T′+Ws′+∫−D/2D/2(u1′)dD·L+Wp  =∫−D/2D/2(u2′)dD·L+N′,

where the superscript sign ( ′) represents the corresponding forces or stresses during shaking of pipe 1. Subtracting (1) from (5) gives

(6) $$\begin{array}{c} \Delta T=\overset{D/2}{\underset{-D/2}{\int }}\left(\Delta u_{2}-\Delta u_{1}\right)dD\cdot L+\Delta N-\Delta W_{s}, \end{array}$$

where Δ*T* is the increment of the stabilizing force which can be measured by the load cell. Δ*u*_1_ and Δ*u*_2_ are excess pore pressures around the pipe. ∫_−*D*/2_^*D*/2^(Δ*u*_2_ − Δ*u*_1_)*dD* · *L* is the integral of excess pore pressure during soil liquefaction, labelled as Δ*U*. Δ*W*_*s*_ and Δ*N* are the increments of support force of soil underlying the pipe and the effective weight of soil overlying the pipe, respectively, due to the flowing deformation of soil around the pipe during vibration [22]. The three parts on the right-hand side of (6) can also be calculated by the integral of earth pressure differences around the pipe, marked as Δ*P*.

**Figure 14:**
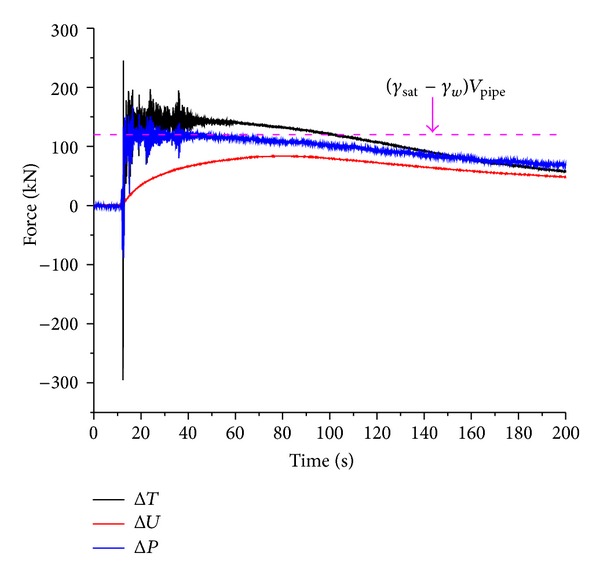
Relationship among Δ*T*, Δ*P* and Δ*U* under 0.5 g El-Centro wave in test 1.

Δ*T*, Δ*P*, and Δ*U* of pipe 2# under 0.5 g excited by El-Centro wave are given in Figure 14. It is clear that the growth patterns of Δ*T* and Δ*P* are almost the same. As the number of earth pressure transducers installed around the pipe is limited, the slight difference between Δ*P* and Δ*T* is reasonable. The integral of the excess pore pressure, Δ*U*, which is defined as the dynamic buoyancy force in this paper, is smaller than both Δ*P* and Δ*T*. Obviously, the uplifting behavior of pipe is not only affected by the build-up of the excess pore pressure, but also by the variations of the effective weight of the overlying soil and the support force of the underlying soil.

The value of (*γ*_sat⁡_ − *γ*_*w*_) · *V*_pipe_, which is commonly adopted by other researchers to calculate the buoyancy force, is given in Figure 14. When the soil is fully liquefied, the excess pore pressure then reaches the value of the initial effective vertical earth pressure (the pore pressure at the crown surface of the pipe is *u*_1_′ = *γ*_sat⁡_ *f*(*h*_1_), and the value on the bottom of the pipe is *u*_2_′ = *γ*_sat⁡_ *f*(*h*_2_)). Integrating the difference between *u*_1_′and *u*_2_′ gives

(7) ∫−D/2D/2(u2′−u1′)dD·L =γsat⁡·∫−D/2D/2[f(h2)−f(h1)]dD·L=γsat⁡Vpipe.

It indicates that the dynamic buoyancy force Δ*U* is equal to (*γ*_sat⁡_ − *γ*_*w*_) · *V*_pipe_ for fully liquefied ground. The value of dynamic buoyancy force will be overestimated for incompletely liquefied soil by (7).

### 4.3. Force Analysis during Shaking for Pipe 1#

**Figure 15:**
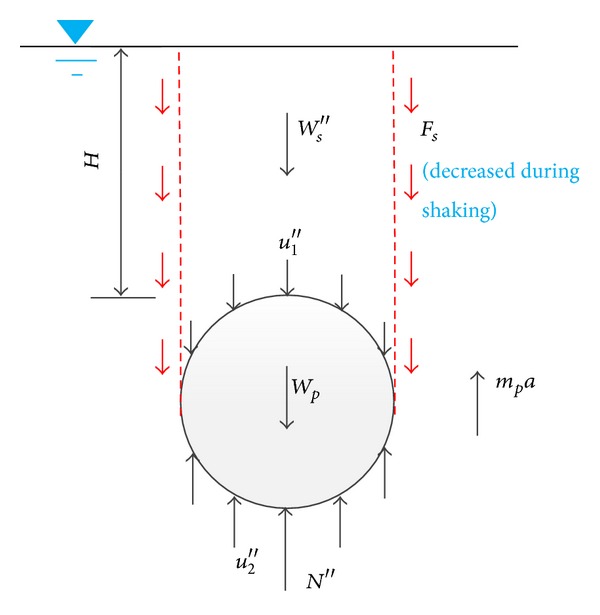
Schematic diagram of force of pipe 1# during shaking.

The force state of pipe 1# during uplifting is shown in Figure 15. And force equilibrium equation is expressed as follows:

(8) Fs+Ws′′+∫−D/2D/2u1′′dD·L+Wp  =∫−D/2D/2u2′′dD·L+N′′+mpa,

where *a* represents the uplift acceleration of pipe; *F*_*s*_ represents the frictional resistance from the overlying soil, which varies with the degree of soil liquefaction and reduces to 0 if the soil is fully liquefied. The physical meanings of *u*_1_′′, *u*_2_′′, *W*_*s*_′′, and *N*′′ are the same as *u*_1_′, *u*_2_′, *W*_*s*_′, and *N*′, except the superscript sign ( ′′  ) represents the corresponding forces or stresses during vibration of pipe 1#. Subtracting (1) from (8) gives

(9) $$\begin{array}{c} m_{p}a=F_{s}+\Delta^{*}W_{s}-\overset{D/2}{\underset{-D/2}{\int }}\left(\Delta^{*}u_{2}-\Delta^{*}u_{1}\right)dD-\Delta^{*}N, \end{array}$$

where Δ*u_1_and Δ*u_2_ are excess pore pressures around the pipe; ∫_−*D*/2_^*D*/2^(Δ*u_2_ − Δ*u_1_)*dD* · *L* is dynamic buoyancy force during soil liquefaction, labelled as Δ*U. Δ*W_*s*_ and Δ*N are the increments of support force of soil underlying pipe 1# and the effective weight of soil overlying pipe 1#, respectively, due to the excess pore pressure variation induced flowing deformation of soil around the pipe during vibration. As the different motion patterns of pipe 1# and pipe 2#, the stresses measured by the transducers around them are different. The total force of the three parts can also be calculated by the integral of earth pressure around pipe 1#. All the forces in (9) can be calculated by the measured data except *F*_*s*_.

The frictional force *F*_*s*_ from the shear plane is estimated using the equation introduced by DNV (2007) [27] as follows:

(10) $$\begin{array}{c} F_{s}=f_{p}\left[\frac{D}{H}\times {\left(\frac{H}{D}+0.5\right)}^{2}\right]\sigma_{H}^{\prime }DL, \end{array}$$

where *σ*_*H*_′ refers to the effective vertical stress of the overlying soil. Before vibration, *σ*_*H*0_′ = *γ*′*H*. *f*_*p*_ is a parameter related to the soil property ranging from 0.4 to 0.6 for medium dense sand.

Given the relationship between the degree of liquefaction and the frictional contact between the soil grains, vertical effective stress declines linearly with the increase of excess pore pressure. Consider

(11) $$\begin{array}{c} \sigma_{H}^{\prime }=\sigma_{H0}^{\prime }-\Delta u. \end{array}$$

**Figure 16:**
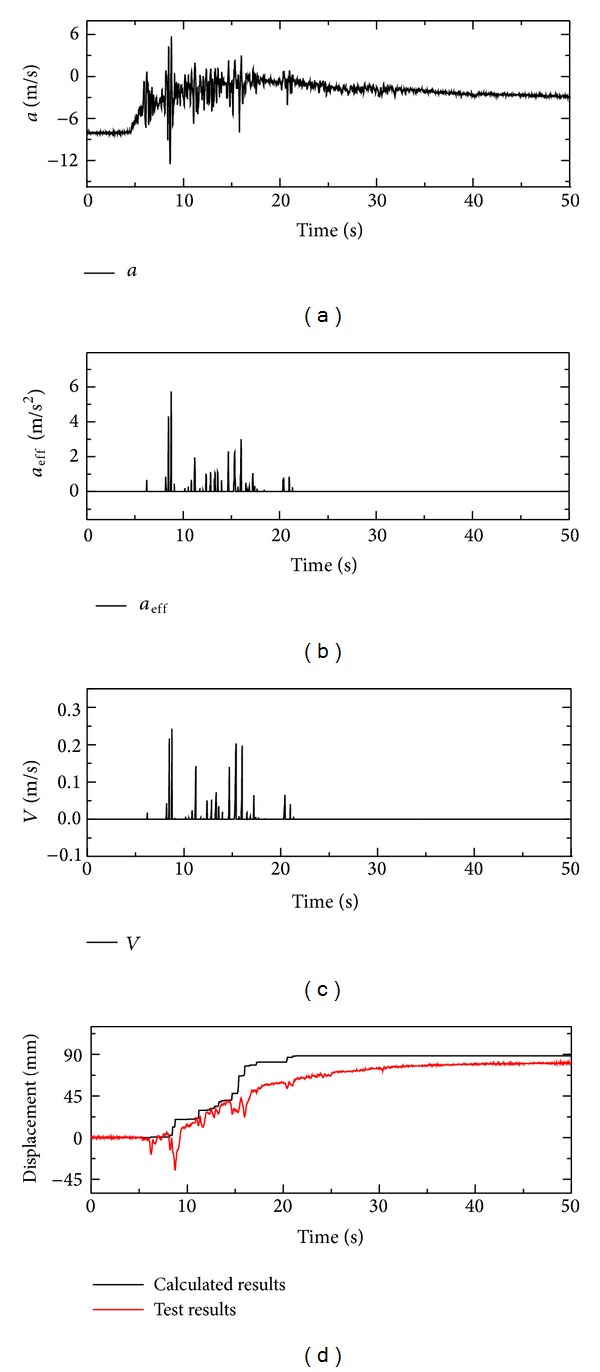
Acceleration, velocity and displacement of pipe 1# under 0.4 g Taft wave in test 1.

Substituting (10) and (11) into (9), the uplift acceleration (*a*) can be obtained. And acceleration time-history is shown in Figure 16(a). The concept of the Newmark's method which is used to predict earthquake-induced permanent deformation is adopted in the case of a floating pipe [28]. The angle of the sliding surface is considered to be vertical rather than inclined. And the rigid pipe can be treated as sliding block of the Newmark's model with vertical movement.

As the behavior of pipe in liquefied soil is extremely complicated, a few assumptions are made in the following to calculate the displacement of pipe. The uplifting of the pipe 1# occurs as soon as the value of the uplift acceleration is greater than zero. Zero is deemed as the yield acceleration. The movement of pipe occurs when its acceleration exceeds the yield acceleration, which is in accordance with Newmark's analysis. The excess in acceleration above yield acceleration is termed as effective vertical acceleration (*a*_eff_). And effective vertical acceleration time-history is illustrated in Figure 16(b). In addition, pipe is incapable of sinking considering the bearing capacity of the underlying soil and the flowing of soil from the top or side to the bottom of the pipe. As pipe 1# is in the static status before shaking, the initial value of acceleration, velocity, and displacement should be zero.

Based on the assumptions above, the accumulated uplifting displacement can be obtained by integrating the effective vertical acceleration *a*_eff_ twice. The uplifting displacement time history of the pipe 1# calculated under 0.4 g Taft wave in test 1 is shown in Figure 16(d). The predicted displacements are slightly larger than the experimental ones observed in Figure 16 and fluctuate around the experimental ones in other cases. The difference between the measured and the predicted displacements is lower than 20 mm, which is deemed to be acceptable as the number of transducers installed around the pipe is limited. And the motion patterns of them are almost the same. Therefore, the proposed approach to estimating the stabilizing force around pipe is reasonable.

## 5. Conclusions

The uplifting behavior of shallow buried pipe in liquefied field was investigated through dynamic centrifuge model tests in the present study, and the main conclusions of the research are summarized as follows.

1. Although the uplifting phenomenon of pipelines in the liquefied soils always happens during the seismic vibration, the observation in our tests shows the begin and end time point of uplifting is not directly related to the seismic motion. The uplifting is highly dependent on the buildup of the excess pore pressure. Moreover, the quantitative relationship between the uplifting behavior and the generation of the excess pore pressure needs further studies.
2. The uplifting movement of pipe is the combination effects of multiple forces. During seismic vibration, excess pore pressure generates and soil around the pipeline gradually flow in an oval-like trace, which causes both the variation of effective weight of overlying soil and supporting force of soil underlying the pipeline, as well as the shear resistance from shear planes that varies with the degree of liquefaction. As a result, the equilibrium of pipeline during shaking is broken and the pipe consequently uplifts. However, in most existing research, the variations of the overlying soil weight and the supporting force of the underlying soil are ignored.
3. For incompletely liquefied field, the buoyancy force is overestimated by multiplying the saturated unit weight of soil and pipeline volume.

## Figures and Tables

**Table 1:** Centrifuge testing program and uplifting status of pipe 1# during tests.

Test number	Seismic excitation			
	Seismic wave	Duration (s)	Amplitude (g)	Uplifting status
Test 1	Noise	30	0.02	
	Zhejiang seism wave		0.1	Remain still
	EL-Centro		0.1	Remain still
	Noise		0.02	
	Zhejiang seism wave		0.15	Sink slightly
	EL-Centro		0.15	Rise slightly
	Noise		0.02	
	EL-Centro		0.5	Rise
	Taft		0.4	Rise
	Noise		0.02	

Test 2	Noise	30	0.02	
	EL-Centro		0.1	Remain still
	Zhejiang seism wave		0.1	Remain still
	Noise		0.02	
	EL-Centro		0.4	Rise
	Taft		0.4	Rise
	Noise		0.02	
